# “Think from the perspective of the reader” and other insiders’ perspectives for scholars in isolation

**DOI:** 10.1007/s40037-020-00629-6

**Published:** 2020-11-17

**Authors:** Erik W. Driessen

**Affiliations:** Maastricht University, Maastricht, The Netherlands

*“Think from the perspective of the reader”* was Kevin Eva’s insider’s perspective for me; a brand-new editor-in-chief. At that time, I couldn’t imagine the impact the advice would have on my work as an editor. For example, I use *“Think from the perspective of the reader”* as a heuristic when confronted with complex editorial situations: what to do with conflicting reviews, or whether or not to allow more words for a manuscript. Kevin’s advice, shared with me over coffee at an international research meeting, was based on his experiences as the editor of *Medical Education.* The advice discussed in this casual conversation opened the window to an insiders’ world (i.e. editors world) that had been unknown to me.

Advice from insiders in health professions education can help you with different aspects of your scholarly life. *“Think from the perspective of the reader”* (Eva) guided my first steps into unknown territory; *“don’t accept an invitation as a speaker on a topic you’re not familiar with”* (van der Vleuten) could have helped me prevent situations I better had avoided; *“one should return from a conference better dressed and better rested”* (Lingard) has helped me balance life and work during conferences. What these insiders’ tips have in common is that they synthesise and make explicit the tacit knowledge gained in many years of experience as an insider. Most advice is about dilemmas or situations with conflicting interests or motivations. For example, the interests of an author (having the chance to use enough words to tell all the details of her study) versus the interests of a reader (concise and clear writing). Such advice can help newcomers find their way in the wonderful, yet sometimes also frightening world of health professions education scholarship. Often these helpful pieces of insider knowledge are shared during casual conversations over coffee or dinners during conferences or other meetings.

The pandemic has brought the conferences, meetings and dinners to a grinding halt. These days we do our scholarly work in isolation. We rarely have these casual conversations with an insider of what is to many of us a new world. This inspired *Perspectives on Medical Education* to initiate a new section: An insider’s perspective for health professions education scholars. We looked for and found an insider in our field willing to share his experiences and wisdom. Dr Glenn Regehr, from the Centre for Health Education Scholarship, University of British Columbia, Vancouver, Canada, will offer his thoughts, contemplations and advice on readers’ dilemmas or questions. Glenn has over 25 years of experience as a scholar in health science education and recently, the Karolinska Prize Committee stressed that *“he has also provided outstanding support and guidance to junior researchers.”* (Professor Sari Ponzer, Chair of the Karolinska Prize Committee). In our view, this makes an excellent insider.

You can send your questions or dilemmas to lieda.meester@bsl.nl. And who knows, your question may be the topic of the next Insider’s Perspective instalment.

In this December edition, Glenn offers his perspective on the dilemma: Should I work on a program of research or can I be more opportunistic in my selection of questions to explore? [1] I hope you’ll enjoy reading this and future Insider’s Perspectives, and that it will help you as much as some insider’s perspectives have helped me (or could have helped me).
